# Should we transfer poor quality embryos?

**DOI:** 10.1186/s40738-020-00072-5

**Published:** 2020-02-19

**Authors:** Anastasia Kirillova, Sergey Lysenkov, Maria Farmakovskaya, Yulia Kiseleva, Bella Martazanova, Nona Mishieva, Aydar Abubakirov, Gennady Sukhikh

**Affiliations:** 1 Federal State Budget Institution “National Medical Research Center for Obstetrics, Gynecology and Perinatology named after Academician V.I. Kulakov of the Ministry of Healthcare of Russian Federation”, st. Academician Oparin 4, 4117513 Moscow, Russia; 2 Department of Evolutionary Biology, Biological Faculty, Moscow State University, Leninskie Gory 1/12, 119991 Moscow, Russia

**Keywords:** Embryo morphology, Ivf, Live birth rate, Poor embryo quality, Blastocyst morphology

## Abstract

**Background:**

To evaluate if it is safe and effective to transfer poor quality embryos.

**Methods:**

It was a retrospective analysis using individual patient data with positive controls. All patients undergoing embryo transfers of poor quality embryos on day 3 or on day 5 as part of fresh In Vitro Fertilization (IVF) cycles performed between 2012 and 2016. This study assessed a total of 738 poor quality embryos from 488 IVF programs. 261 embryo transfers were performed on day 3 (402 embryos were transferred) and 227 on day 5 (336 embryos were transferred). Control group consisted of 9893 fair and good quality embryos from 5994 IVF programs. Outcome rates were compared with two-tailed Fisher exact test using fisher.test function in R software. 95% confidence intervals for proportions were calculated using the Clopper-Pearson method with binom.test function in R. The groups of patients with poor vs. good and fair quality embryos were compared by age, body mass index(BMI), number of oocytes, female and male main diagnosis, cycle type, controlled ovarian stimulation (COS) protocol, the starting day of gonadotropin administration, the starting dose of gonadotropins, the total dose of gonadotropins, the total number of days of gonadotropins administration, the starting day of gonadotropin-releasing hormone (GnRH) agonist administration, the total number of ampoules of GnRH-agonist used, day of the trigger of ovulation administration and the type of the trigger of ovulation using the Student’s t-test for interval variables and with the chi-square test for nominal variables.

**Results:**

No significant differences in the implantation rate, clinical pregnancy rate, miscarriage rate, live births, and the number of children born were found between the groups of poor quality embryos transferred on day 3 and day 5. Though the implantation rate was lower for the group of poor quality embryos, than for the control (13.9% vs 37.2%), statistically significant differences between the proportion of implanted embryos which resulted in clinical pregnancies and live births in both groups were not observed (72% vs 78.2 and 55.8% vs 62.0% respectively).

**Conclusion:**

Transfer of poor quality embryos at either day 3 or day 5 have a low potential for implantation, though those embryos which successfully implanted have the same potential for live birth as the embryos of fair and good quality. This study supports that it is safe to transfer poor quality embryos when they are the only option for fresh embryo transfer (ET).

## Background

Standard morphology assessment has always been the major tool for selecting the best embryo for transfer, and good embryo morphology remains one of the main predictors for the successful outcome of the IVF programs. However, with the development of preimplantation genetic screening, we came to the realization that the current morphological analysis cannot be relied on for choosing an euploid embryo. Fragouli and coauthors have shown that there is no correlation between chromosomal status and morphology score at the cleavage stage. At the blastocyst stage, there is a higher probability of euploidy among good morphology blastocysts, yet still, the effect of aneuploidy on the embryo quality is rather subtle [1]. Even embryos with top morphology can carry genetic abnormalities, and, conversely, there are euploid embryos among those of poor morphology [1–4]. Despite the fact that preimplantation genetic screening (PGS) can improve the IVF outcomes for certain categories of patients [3, 5, 6], it is an invasive method which requires high resources in terms of time and money from couples [7], and for the overall patient population, it does not increase ongoing pregnancy rates [8, 9].

Nowadays, the majority of transfers are performed either on day 3 at the cleavage stage, or on day 5 at the blastocyst stage, and it is advised not to transfer more than 2 embryos at once to minimize the risk of complications in both the mothers and the fetuses [10, 11]. At both stages, embryos can be described as good, fair, or poor depending on their morphological appearance. In general, embryos of poor morphology are not favored to be selected for the embryo transfer, nor for the cryopreservation, and thus are often discarded. This decision relies on the fact that the implantation potential of poor quality embryos is much lower than of good or fair quality. Moreover, there is a belief among specialists that transferred poor quality embryos are likely to lead to spontaneous abortions and miscarriages. However, there is no data supporting this suggestion.

The actual probability of the successful IVF cycles of poor quality embryos has not been thoroughly studied. Moreover, there is no unified guideline available for the fate decision of such embryos. It is also unclear which developmental stage it is more advisable to perform such embryo transfers. In this work we tried to shed light on this problem, analyzing the outcomes of 488 cycles, in which poor quality embryos were transferred, over 5 years. The aim of our study was to evaluate the potencies of embryos of poor morphology to pregnancies and live births and to compare the outcomes of embryo transfers of low quality embryos on day 3 and on day 5.

## Methods

### Study group

This study was carried out on the data collected between January 2012 and December 2016 at the department of reproduction at the National Medical Research Center for Obstetrics, Gynecology and Perinatology. We retrospectively evaluated in vitro fertilization cycles with or without intracytoplasmic sperm injection (ICSI) involving fresh embryo transfers of poor quality embryos on day 3 or on day 5. The allocation of the day of embryo transfer and stage of embryo development for the embryo transfer was assigned randomly. There was a tendency to transfer embryos on day 3 when there were less than 3 embryos available and for patients with several unsuccessful ART treatments in the anamnesis.

The factors of infertility of the female patients were: tubal factor, uterine factor, endocrine factor, polycystic ovary syndrome (PCOS), diminished ovarian reserve, endometriosis, multiple factors and other. Other factors included obesity, human immunodeficiency virus (HIV) and hepatitis infections, thrombophilia, and chromosomal abnormalities. The male fertility parameters were subdivided into 4 categories: normozoospermia, subfertile (oligo-, asteno- or oligoastenozoospermia), cryprtozoospermia and other. The last category included male patients with such factors of infertility as varicocele, HIV and hepatitis infections, and chromosomal abnormalities. If donor gametes were used that then the patients’ diagnosis was not taken into consideration and assigned to the group “normal”.

The main outcome measures were: implantation rate, biochemical pregnancy rate, clinical pregnancy rate, miscarriage rate, and live birth rate. A total of 738 poor quality embryos from 488 IVF programs were included. 261 embryo transfers were performed on day 3 (402 embryos were transferred) and 336 on day 5 (227 embryos were transferred). First, clinical outcomes between poor quality embryos transferred on day 3 and day 5 were compared in order to determine which developmental stage was more favorable for ET. Then all cases of poor quality embryos ET were grouped and compared against ET with fair/good quality embryos (the control group). The control group consisted of 9893 embryos of good or fair quality from 5994 IVF programs.

Several patients contributed several times to the analyses.

In the group with poor quality embryos, 2 couples participated twice in the study. In the control group, 765 couples contributed twice to the analyses, 160 couples – 3 times, 48 couples 4 times, 16 couples 5 times, 5 couples 6 times and 1 couple 7 times. We treated each IVF program as the new treatment since the pool of oocytes and the oocyte quality is different in each menstrual cycle, and embryo quality can differ between IVF programs of one patient.

Informed consent summarizing the IVF, ICSI, extended culture, embryo vitrification, and the possible inclusion of their embryos in the research program was obtained from all patients.

### Ovarian stimulation

Ovarian stimulation was performed with the GnRH-agonists and GnRH-antagonists (Cetrorelixum, Merck Serono, SA, Switzerland, 0.25 mg) protocols. Patients injected an individually selected dose of follicle-stimulating hormone and luteinizing hormone (Menopur; Ferring; Puregon; Organon, the Netherlands or Gonal-F; Serono, Switzerland) in the range of 75–200 IU per day till the day of administration of trigger of ovulation. In the course of ovarian stimulation, the doses of gonadotropins were adjusted according to the individual patient’s ovarian response. For the triggering of ovulation, we used hCG human chorionic gonadotropin (Pregnyl, MSD, the Netherlands; Ovitrelle; Merck, Switzerland or Сhoragon; Ferring; Germany) and/or triptorelin (Diphereline; Ipsen Pharma) when at least three follicles measured ≥17 mm in diameter. Ultrasound-guided oocyte retrieval was conducted 36 h later.

### Fertilization and embryo culture

Semen samples were evaluated according to the World Health Organization guidelines. Fertilization check was performed 16–18 h after fertilization. The presence of two clearly distinctive pronuclei (2pn) determined the successful fertilization. Embryos were cultured in ISM1 and Blast Assyst (Origio, Denmark) with the change of medium on day 3.

### Morphological evaluation

Cleavage stage embryos were evaluated based on the following parameters: cell number, evenness of the blastomeres, amount of cellular fragmentation, and the presence of multiple micronuclei or cytoplasmic abnormalities. The grade from 1 to 3 was assigned to each embryo according to the Istanbul consensus scoring system for cleavage-stage embryos [12].

On day 5 assessments were performed according to Gardner classification. The score was given according to the degree of expansion and hatching status (from 1 to 6) and the amount of cells in inner cell mass (ICM) and trophectoderm (TE) (from A to C).

In our study embryos were considered to be of poor quality if they had grade 3 on day 3 and had C score for both ICM and TE, or failed to form a blastocyst at all on day 5. Embryos with grades 1 or 2 on day 3 and embryos which had at least 1 score A or B for ICM or TE (AA, AB, BA, BB, BC, and CB) on day 5 were scored as fair and good quality embryos, and served as control.

### Pregnancy outcomes

Biochemical pregnancy was confirmed by a plasma beta-hCG concentration > 10 IU/l on day 14 after ET. Clinical pregnancy was defined from the ultrasonographical presence of a gestational sac at 2 weeks after positive beta-hCG results. A pregnancy loss earlier than the 22nd gestational week was considered a miscarriage.

### Statistical analysis

Outcome rates were compared with the two-tailed Fisher exact test using fisher.test function in R software (R Core Team, 2016). 95% confidence intervals for proportions were calculated using Clopper-Pearson method with binom.test function in R. The groups of patients were compared with the Student’s t-test for interval variables and with the chi-square test for nominal variables.

## Results

This study includes the retrospective analyses of IVF cycles involving embryo transfers of poor quality embryos on day 3 and day 5. We assessed a total of 488 transfers of poor quality embryos: 261 were performed on day 3 (mean number of embryos per transfer - 1.35 ± 0.5), and 227 were performed on day 5 (mean number of embryos per transfer - 1.48 ± 0.7). The control group consisted of 5506 embryo transfers (mean number of embryos per transfer - 1.65 ± 0.1). The patients’ characteristics are summarized in Supplementary Tables 1 and 2. The statistical analyses revealed that the analyzed groups are homogeneous. No statistical differences have been found between the groups of patients with poor quality embryos for ET vs the group with good and fair quality for such parameters as age, BMI, number of oocytes and female main diagnosis. The only statistically significant difference was identified in the diagnosis of male partners (Table S2). In the group of patients with transferred poor quality embryos, there was a statistically lower number of mature oocytes (mean number of oocytes 5.4 ± 4.64 vs 6.1 ± 4.22).

The cycle characteristics are reported in Supplementary Tables 1 and 2. The groups of patients with different embryo quality for embryo transfer is comparable. No statistically significant differences have been identified for such parameters as cycle type, COS protocol, the starting day of gonadotropin administration, the starting dose of gonadotropins, the total dose of gonadotropins, the total number of days of gonadotropins administration, the starting day of GnRH-agonist administration, the total number of ampoules of GnRH-agonist used, day of the trigger of ovulation administration and type of the trigger of ovulation.

The difference for the mean number of embryos transferred in patients with poor quality embryos and the control group was identified (1.51 ± 0.5% vs 1.65 ± 0.1% respectively). Though the difference was statistically significant, yet minor.

The main outcome measures in our study were: biochemical pregnancy rate, implantation rate, clinical pregnancy rate, miscarriage rate, and live births. The clinical outcomes obtained in our laboratory in 5 years (2012–2016) are reported in Table 1.

**Table 1:** The clinical outcomes of poor quality embryos transfers

	poor quality embryos		
	day 3 ET	day 5 ET	*p*
No. of patients	261	227	
Female age (years) (95% CI)	34.5 ± 5.40	33.8 ± 5.51	0.1653
No. of transferred embryos	402	336	
Mean number of embryos per ET (95% CI)	1.54 ± 0.06	1.48 ± 0.7	0.186
Biochemical pregnancy rate (95% Cl)	12.3% (32/261)	15.8% (36/227)	0.294
Implantation rate (95% Cl)	6.5% (26/402)	7.4% (25/336)	0.6628
Clinical pregnancy rate (95% Cl)	9.6% (25/261)	10.6% (24/227)	0.764
Live birh rate (95% Cl)	7.7% (20/261)	8.3% (19/227)	0.8703
Miscarriage rate (95% Cl)	1.9% (5/261)	2.6%(6/227)	0.762

**Fig. 1:**
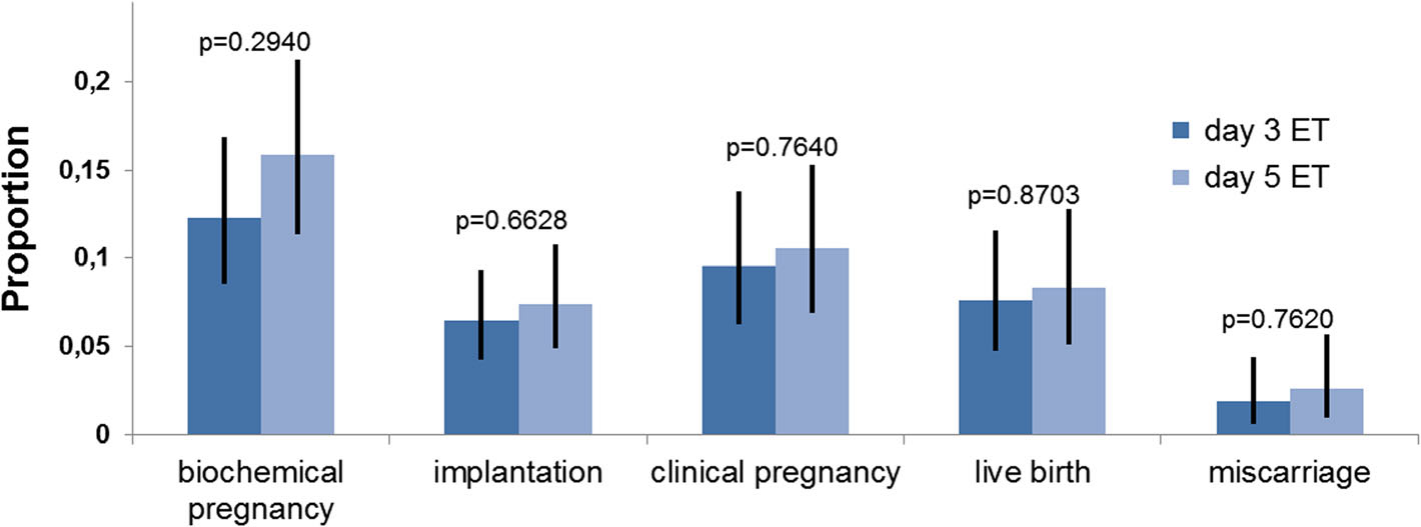
Reproductive outcomes after poor quality embryo transfers on day 3 (Dark blue) and on day 5 (Light blue).*P < 0.001

The clinical outcomes for the day 3 and day 5 embryo transfers were: biochemical pregnancy rate (12.3% vs 15.8% respectively, *p* = 0.294), implantation rate (6.5% vs 7.4% respectively, *p* = 0.6628), clinical pregnancy rate (9.6% vs 10.6%, *p* = 0.764), live birth rate (7.7% vs 8.3%, *p* = 0.8703), and miscarriage rate (1.9% vs 2.6%, *p* = 0.762). Thus, there are no statistical differences in any of the outcomes of the cycles in which embryos of poor morphology were transferred on day 3 or day 5 (Fig. 1). Therefore, these two groups were combined in one group and compared with the control group.

**Fig. 2:**
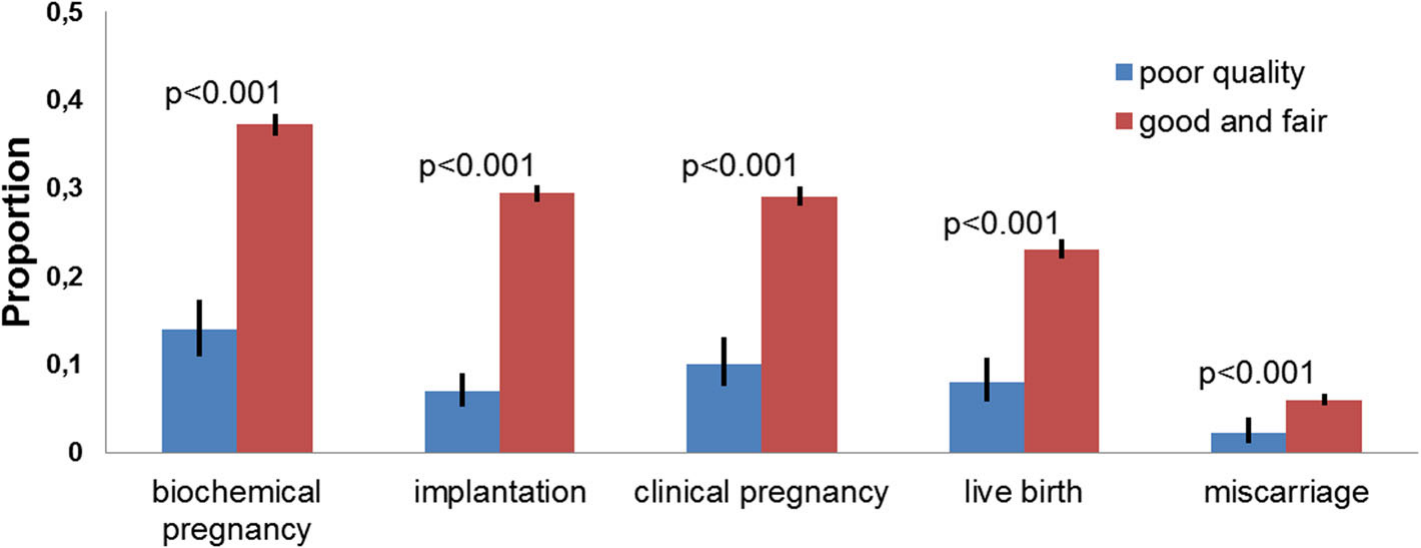
Reproductive outcomes after poor quality embryo transfers (Blue) and transfers of embryos of good and fair quality (Red).*P < 0.001

All clinical outcomes were lower for the group of poor quality embryos than for the control: the biochemical pregnancy rate (13.9% vs 37.2%, *p* < 0.001), implantation rate was (6.9% vs 29.4%, p < 0.001), clinical pregnancy rate (10% vs 29.1%, p < 0.001), live birth rate (8.0% vs 23.1%, p < 0.001), miscarriage rate (2.2% vs 6.0%, p < 0.001) respectively. All poor quality embryos have lower rates of implantation, biochemical and clinical pregnancies and live births (Fig. 2; Table 2).

**Table 2:** Clinical outcomes of poor and good/fair quality embryos

	poor quality	good and fair	*p*
No. of patients	488	5994	
Female age (years) (95% CI)	34,4 ± 6,24	33,9 ± 5,35	0.1478
No. of transferred embryos	738	9893	
Mean number of embryos per ET (95% CI)	1.51 ± 0.5	1.65 ± 0.1	**p < 0.001**
Biochemical pregnancy rate (95% Cl)	13.9% (68/488)	37.2% (2231/5994)	***p*** **< 0.001**
Implantation rate (95% Cl)	6.9% (51/738)	29.4% (2913/9893)	**p < 0.001**
Clinical pregnancy rate (95% Cl)	10% (49/488)	29.1% (1746/5994)	**p < 0.001**
Live birh rate (95% Cl)	8.0% (39/488)	23.1% (1385/5994)	**p < 0.001**
Miscarriage rate (95% Cl)	2.2% (11/488)	6% (361/5994)	**p < 0.001**
Clinical pregnancy rate/ biochemical pregnancy (95% Cl)	72% (49/68)	78.3% (1746/2231)	0.2313
Live birth rate/clinical pregnancy (95% Cl)	79.6% (39/49)	79.3% (1385/1746)	0.9636

**Fig. 3:**
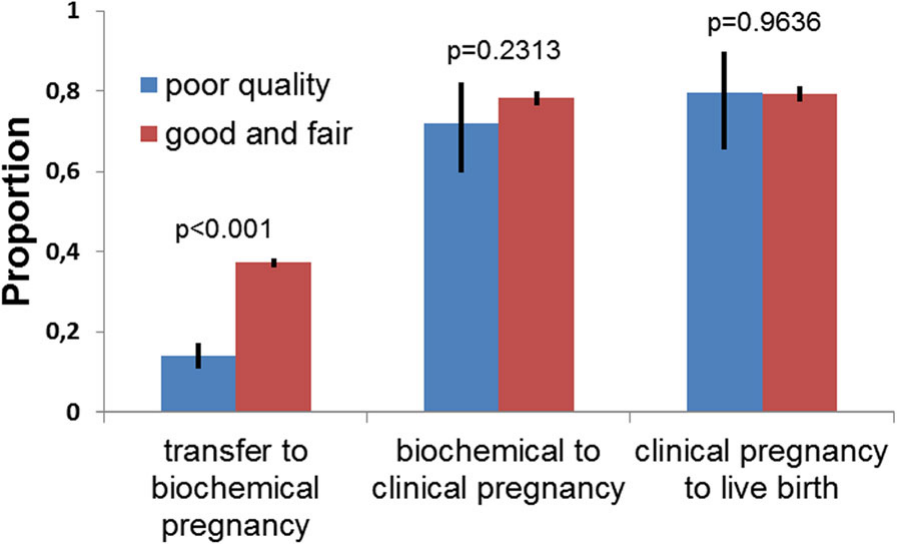
Probabilities of pregnancies development from one sequential stage to another after poor quality embryo transfers (Blue) and transfers of embryos of good

Then we decided to assess the probabilities of embryos of different morphological quality to reach the next sequential stage. In order to do that we compared the portion of biochemical pregnancies leading to the clinical ones, and the portion of clinical pregnancies leading to live births in poor quality and control embryos. Statistically significant differences between the portion of implanted embryos which resulted in clinical pregnancies (72% vs 78.3%, *p* = 0.2313) and the portion of clinical pregnancies which resulted in live births (79.6% vs 79.3% *p* < 0.7222) in both groups were not observed (Fig. 3). Thus, poor quality embryos have lower probability for biochemical pregnancies, but if they implant they have the same potential for development and live birth as fair and good quality embryos.

We compared the group of patients who got pregnant with poor quality embryos with the group of patients who got pregnant with good and fair quality embryos. The statistical analyses revealed that there were no differences in any analyzed parameters. The comparison of groups of patients who did not become pregnant revealed that the only statistically significant difference was in male diagnosis (Table S1 and Table S2).

## Discussion

Taken together, our results show that the IVF outcomes are the same for poor quality embryos transferred either on day 3 or on day 5. The results of the present investigation are in line with the findings of an earlier study by Balaban and coauthors [13] who demonstrated that the implantation rate of poor quality embryos on day 3 and day 5 did not differ (5.9 and 5.2% per embryo). The clinical pregnancy rate in that work was higher for blastocysts of low quality compared to low quality cleavage stage embryos (27.2% vs. 16.6% per embryo transfer). However, that study was conducted on the onset of blastocyst culture protocols and the mean number of embryos transferred was much higher than advised nowadays: 5.2 on day 3 and 2.4 embryos on day 5.

Since there is no difference in the outcome of poor quality embryo transfers on day 3 or on day 5, we believe that embryos of poor morphology should be dealt with regarding the day of the transfer in the same manner as embryos of good and fair morphology. For the general group of embryos of all morphological grades, the Cohrane study did not find any evidence of the difference in live birth or pregnancy outcomes between day 2–3 and day 5–6 transfers of embryos [14].

Therefore, in order to make a decision on which day to transfer an embryo of poor morphology we think secondary factors, such as a patient’s comfort and convenience, costs, and laboratory burden, should be considered. According to our data, transfers of embryos of low morphology do not increase the rate of spontaneous abortions. The portion of implanted embryos, which resulted in developing pregnancies and live births, did not differ significantly in the treatments with poor quality embryos for ET from the controlled group. Consequently, after implantation, an embryo has an equal potential for development regardless of its morphology at the preimplantation stage.

Statistical analyses revealed that the group of patients with poor quality embryos had a lower number of mature oocytes. This might indicate that in this group there was a smaller range of embryos available for selection for embryo transfer and the patients ended up with embryos of lower quality. Another statistically significant difference was identified in male diagnosis between the groups. However, the differences in values are very subtle and we believe have limited clinical significance. No other parameters of COS nor patients characteristics differed in the groups of comparison. It indicates that embryo quality was the major factor playing the role in clinical outcomes in this study.

Unfortunately, it is extremely difficult to find out which developmental mechanisms play a key role in implantation failure of poor quality embryos since there are limited tools for studying human embryo development beyond one week post fertilization. We might argue that the low amount of trophectoderm cells plays a key role in this process. As indirect evidence to this, it has recently been shown that trophectoderm biopsy is associated with the decreased implantation rates due to its possible effect on the proper placental development and function [15, 16].

Moreover, it is likely that the poor quality embryos fail to implant due to the chromosome abnormalities they carry. It has been demonstrated by Capalbo and coauthors [17] that if an embryo is euploid, it has the same implantation potential regardless of its morphology and developmental rate.

Interestingly, there was a higher miscarriage rate in treatments with good and fair quality embryos compared to the programs with poor quality embryos for ET. We speculate that more aneuploid embryos of good and fair quality are able to implant due to the superior trophectoderm morphology, while poor quality embryos fail to do that. Consequently, such aneuploid embryos of higher quality might result in a higher miscarriage rate.

There are some limitations to this study. It is a retrospective unicenter study and not all COS and patients characteristics were available for all patients. Another major limitation is that there was a statistically significant difference for the mean number of embryos transferred in patients with poor quality embryos and good and fair quality embryos. Also, some patients contributed several times to the study, and each IVF treatment was considered separately.

## Conclusions

Our data indicate that the general outcomes of the IVF cycles where poor morphology embryos were transferred were lower only due to the decreased implantation potential of such embryos. Thus, our results suggest that it is safe to transfer poor quality embryos as there are no higher risks of miscarriages or stillbirths compared to the transfers of embryos of good or fair morphology. It has been previously shown that embryos of poor quality do not increase the rate of chromosomal abnormalities, congenital malformations of children born, or perinatal complications and mortality [18, 19]. Therefore, we strongly advise against discarding embryos of poor quality when they are the only option for fresh embryo transfer. Even though the implantation rate of such embryos is lower than of good quality embryos, it would still be higher than canceling embryo transfer at all. Our practice transfers of poor morphology embryos resulted in the birth of 40 children.

## Supplementary information

**Additional file 1.Supplementary Table 1**. Protocols of COS and patients' characteristics.

**Additional file 2.Supplementary Table 2**. Protocols of COS and patients' characteristics.

## Data Availability

The datasets analysed during the current study are available from the corresponding author on reasonable request.

## Abbreviations

COS: Controlled ovarian stimulation
G&D QE: Good and fair quality embryos
GnRH: Gonadotropin Releasing Hormone
HIV: Human immunodeficiency virus
ICM: Inner cell mass
ICSI: Intracytoplasmic sperm injection
IVF: In vitro fertilization
PGS: Preimplantation genetic screening
PQE: Poor qaulity embryos
TE: Trophectoderm
y.o.: years old
