# Supplementary material for: Should we transfer poor quality embryos?
Source: Fertil Res Pract. 2020 Feb 19;6:2. doi: 10.1186/s40738-020-00072-5 (PMC7031982; doi:10.1186/s40738-020-00072-5)
Supplement: Supplementary file 1 — Additional file 1.Supplementary Table 1. Protocols of COS and patients' characteristics. [file 40738_2020_72_MOESM1_ESM.docx]

|  | **all** | | | **pregnant** | | | **nonpregnant** | | |
| --- | --- | --- | --- | --- | --- | --- | --- | --- | --- |
|  | **G&F QE** | **PQE** | ***p*** | **G&F QE** | **PQE** | ***p*** | **G&F QE** | **PQE** | ***p*** |
| Female age (95% CI) | 33.9 ±5.35 (N=5994) | 34.4 ±6.24 (N=496) | p=0.148 | 32.9 ±4.90 (N=1746) | 34.0 ±10.51 (N=49) | p=0.489 | 34.4 ±5.46 (N=4248) | 34.4 ±5.59 (N=447) | p=0.890 |
| BMI (95% CI) | 23.4±4.45 (N=4349) | 23.4 ±3.99 (N=381) | p=0.860 | 23.3±4.27 (N=1245) | 23.2 ±3.01 (N=39) | p=0.828 | 23.4±4.52 (N=3104) | 23.5 ±4.09 (N=343) | p=0.899 |
| starting day of gonadotropins administration (95% CI) | 2.2±1.41 (N=5260) | 2.1 ±1.42 (N=425) | p=0.027 | 2.4±1.45 (N=1575) | 2.3 ±1.56 (N=45) | p=0.726 | 2.2±1.38 (N=3685) | 2.2 ±1.40 (N=380) | p=0.095 |
| starting dose of gonadotropins (95% CI) | 234.3±77.45 (N=4647) | 242.4 ±77.46 (N=355) | p=0.057 | 222.1±73.38 (N=1453) | 240.2 ±82.34 (N=42) | p=0.138 | 239.4±78.71 (N=3194) | 242.7 ±74.63 (N=313) | p=0.479 |
| total dose of gonadotropins (95% CI) | 1909.8±896.63 (N=5308) | 1924.6±936.86 (N=431) | p=0.743 | 1856.5±827.26 (N=1600) | 2027.5±827.26 (N=45) | p=0.172 | 1932.8±924.12 (N=3708) | 1912.6±924.12 (N=386) | p=0.683 |
| total number of days of gonadotropins administration (95% CI) | 8.1±2.76 (N=5537) | 8.1 ±2.88 (N=446) | p=0.752 | 8.3±2.52 (N=1656) | 8.4 ±2.28 (N=46) | p=0.695 | 8.0±2.85 (N=3881) | 8.0 ±2.94 (N=400) | p=0.966 |
| starting day of GnRH-agonis administration (95% CI) | 105.3±3.26 (N=4809) | 105.3±3.37 (N=396) | p=0.057 | 105.4±3.24 (N=1417) | 105.7±3.39 (N=41) | p=0.539 | 105.2±3.27 (N=3392) | 105.3±3.37 (N=355) | p=0.778 |
| total number of ampules of GnRH-agonist used (95% CI) | 4.5±1.79 (N=4944) | 4.6±1.88 (N=403) | p=0.602 | 4.6±1.81 (N=1464) | 4.6±1.91 (N=42) | p=0.902 | 4.5±1.79 (N=3480) | 4.6±1.88 (N=361) | p=0.485 |
| day of trigger of ovulation administration (95% CI) | 10.9±2.53 (N=5586) | 10.8±2.54 (N=466) | p=0.404 | 11.1±2.50 (N=1645) | 10.9±1.77 (N=47) | p=0.719 | 10.9±2.54 (N=3941) | 10.8±2.61 (N=419) | p=0.649 |
| number of oocytes (95% CI) | 7.6±5.14 (N=5993) | 5.14±5.70 (N=496) | p=0.005 | 8.7±5.04 (N=1745) | 6.6±4.22 (N=49) | p=0.002 | 7.1±5.10 (N=4248) | 6.9±5.84 (N=447) | p=0.602 |
| number of MII oocytes (95% CI) | 6.1±4.22 (N=5974) | 5.4±4.64 (N=496) | **p<0.001** | 7.1±5.3 (N=1743) | 5.3±3.77 (N=49) | p=0.003 | 5.7±4.16 (N=4231) | 5.4±4.73 (N=447) | p=0.198 |
