# Supplementary material for: Should we transfer poor quality embryos?
Source: Fertil Res Pract. 2020 Feb 19;6:2. doi: 10.1186/s40738-020-00072-5 (PMC7031982; doi:10.1186/s40738-020-00072-5)
Supplement: Supplementary file 2 — Additional file 2.Supplementary Table 2. Protocols of COS and patients' characteristics. [file 40738_2020_72_MOESM2_ESM.docx]

|  | **all** | | | **pregnant** | | | **nonpregnant** | | |
| --- | --- | --- | --- | --- | --- | --- | --- | --- | --- |
|  | **G&F QE** | **PQE** | ***p*** | **G&F QE** | **PQE** | ***p*** | **G&F QE** | **PQE** | ***p*** |
| **Type of cycle** |  | | | | | | | | |
| ICSI | 55% (3279) | 55% (275) | p=0.383 | 54% (943) | 67% (33) | p=0.762 | 55% (2336) | 54% (242) | p=0.526 |
| ICSI-OD | 3% (197) | 3% (16) |  | 3% (51) | 4% (2) |  | 3% (146) | 3% (14) |  |
| ICSI-OSD | 0% (4) | 0% (0) |  | 0% (2) | 0% (0) |  | 0% (2) | 0% (0) |  |
| ICSI-PGD | 2% (100) | 3% (14) |  | 1% (15) | 0% (0) |  | 2% (85) | 3% (14) |  |
| ICSI-SD | 2% (110) | 1% (4) |  | 1% (26) | 0% (0) |  | 2% (84) | 1% (4) |  |
| IVF | 33% (1977) | 32% (158) |  | 35% (614) | 22% (11) |  | 32% (1363) | 33% (147) |  |
| IVF-OD | 0% (24) | 0% (0) |  | 1% (9) | 0% (0) |  | 0% (15) | 0% (0) |  |
| IVF-OSD | 0% (3) | 0% (0) |  | 0% (2) | 0% (0) |  | 0% (1) | 0% (0) |  |
| IVF-PGD | 0% (4) | 0% (0) |  | 0% (0) | 0% (0) |  | 0% (4) | 0% (0) |  |
| IVF-SD | 3% (183) | 3% (16) |  | 3% (49) | 4% (2) |  | 3% (134) | 3% (14) |  |
| IVF+ICSI | 2% (113) | 3% (13) |  | 2% (35) | 2 %(1) |  | 2% (78) | 3% (12) |  |
| **Female diagnosis** |  | | | | | | | | |
| diminished ovarian reserve | 26% (1457) | 32% (149) | p=0.751 | 18% (290) | 29% (14) | p=0.485 | 29% (1167) | 32% (135) | p=0.383 |
| tubal factor | 35% (1967) | 31% (147) |  | 39% (633) | 38% (18) |  | 34% (1334) | 31% (129) |  |
| endometriosis | 7% (404) | 7% (33) |  | 8% (124) | 8% (4) |  | 7% (280) | 7% (29) |  |
| endocrine factor | 4% (203) | 3% (12) |  | 4% (68) | 2% (1) |  | 3% (135) | 3% (11) |  |
| PCOS | 4% (246) | 5% (25) |  | 5% (78) | 2% (1) |  | 4% (168) | 6% (24) |  |
| uterine factor | 5% (273) | 5% (25) |  | 5% (86) | 8% (4) |  | 5% (187) | 5% (21) |  |
| multiple factors | 1% (29) | 0% (0) |  | 1% (9) | 0% (0) |  | 1% (20) | 0% (0) |  |
| other factors | 1% (56) | 1% (7) |  | 1% (17) | 0% (0) |  | 1% (39) | 2% (7) |  |
| normal | 17% (969) | 15% (71) |  | 20% (325) | 13% (6) |  | 16% (644) | 15% (65) |  |
| **Male diagnosis** |  | | | | | | | | |
| normozoospermia | 41% (2176) | 40% (172) | **p<0.001** | 40% (613) | 31% (13) | p=0.009 | 42% (1563) | 41% (159) | **p=0.001** |
| subfertile | 53% (2779) | 52% (226) |  | 55% (846) | 55% (23) |  | 52% (1933) | 52% (203) |  |
| cryptozoospermia | 6% (291) | 7% (29) |  | 5% (12) | 12% (5) |  | 6% (206) | 6% (24) |  |
| other factors | 0% (7) | 1% (5) |  | 0% (3) | 2% (1) |  | 0% (4) | 1% (4) |  |
| **Protocol** |  | | | | | | | | |
| GnRH-agonists | 8% (448) | 7% (34) | p=0.017 | 9% (154) | 8% (4) | p=0.777 | 7% (294) | 7% (30) | p=0.185 |
| GnRH-antagonists | 85% (5008) | 84% (406) |  | 87% (1496) | 86% (42) |  | 85% (3512) | 83% (364) |  |
| Other protocols | 5% (276) | 8% (38) |  | 2% (34) | 4% (2) |  | 6% (242) | 8% (36) |  |
| Natural cycle | 2% (137) | 2% (8) |  | 2% (40) | 2% (1) |  | 2% (97) | 2% (7) |  |
| **Trigger** |  | | | | | | | | |
| Pregnyl | 69% (3620) | 70% (305) | p=0.974 | 70% (1068) | 74% (35) | p=0.881 | 69% (2552) | 70% (270) | p=0.593 |
| Ovitrelle | 2% (88) | 2% (9) |  | 2% (31) | 2% (1) |  | 2% (57) | 2% (8) |  |
| Choragon | 16% (861) | 14% (62) |  | 16% (251) | 15% (7) |  | 16% (610) | 14% (55) |  |
| Diphereline | 13% (672) | 13% (58) |  | 12% (183) | 9% (4) |  | 13% (489) | 14% (54) |  |
